# Estrogen Receptor β as a Candidate Regulator of Sex Differences in the Maternal Immune Activation Model of ASD

**DOI:** 10.3389/fnmol.2021.717411

**Published:** 2021-08-31

**Authors:** Madeline L. Arnold, Kaoru Saijo

**Affiliations:** ^1^Department of Molecular and Cell Biology, University of California, Berkeley, Berkeley, CA, United States; ^2^Helen Wills Neuroscience Institute, University of California, Berkeley, Berkeley, CA, United States

**Keywords:** estrogen receptor β, brain myeloid cells, maternal immune activation, autism spectrum disorder, sex differences, inflammation

## Abstract

Interestingly, more males are diagnosed with autism spectrum disorder (ASD) than females, yet the mechanism behind this difference is unclear. Genes on the sex chromosomes and differential regulation by sex steroid hormones and their receptors are both candidate mechanisms to explain this sex-dependent phenotype. Nuclear receptors (NRs) are a large family of transcription factors, including sex hormone receptors, that mediate ligand-dependent transcription and may play key roles in sex-specific regulation of immunity and brain development. Infection during pregnancy is known to increase the probability of developing ASD in humans, and a mouse model of maternal immune activation (MIA), which is induced by injecting innate immune stimulants into pregnant wild-type mice, is commonly used to study ASD. Since this model successfully recaptures the behavioral phenotypes and male bias observed in ASD, we will discuss the potential role of sex steroid hormones and their receptors, especially focusing on estrogen receptor (ER)β, in MIA and how this signaling may modulate transcription and subsequent inflammation in myeloid-lineage cells to contribute to the etiology of this neurodevelopmental disorder.

## Introduction

Many neurodevelopmental disorders (NDDs), such as autism spectrum disorder (ASD), attention-deficit/hyperactivity disorder (ADHD), and schizophrenia, show sex differences (Waddell and McCarthy, 2012; Hanamsagar and Bilbo, 2016; Hill, 2016; McCarthy, 2016; Bordeleau et al., 2019; May et al., 2019; Lord et al., 2020; Merikangas and Almasy, 2020); yet the mechanisms behind these observations are poorly understood. For example, it is known that males are more frequently diagnosed with ASD than females (Baron-Cohen et al., 2011; Loomes et al., 2017; Dietz et al., 2020). Several studies indicate a male to female ratio of approximately 3:1 or 4:1 in ASD, as well as sex differences in symptoms (Loomes et al., 2017; Hull et al., 2020). To explain this sex difference in ASD, several hypotheses have been proposed. One possibility is that sex chromosome gene effects contribute to ASD etiology. Indeed, mutations in many genes are known to increase the probability of ASD, and some of them, such as *FMR1*, *MeCP2*, and neuroligins 3 and 4, are on the X-chromosome (Marco and Skuse, 2006; Guy et al., 2011; Percy, 2011; Zhang et al., 2017; Sledziowska et al., 2020; Savatt and Myers, 2021). While it will not be addressed here, excellent reviews that discuss the chromosomal contributions to sex differences in ASD can be found elsewhere (Marco and Skuse, 2006; Guy et al., 2011; Percy, 2011; Zhang et al., 2017; Sledziowska et al., 2020; Savatt and Myers, 2021). Another possible explanation for the sex differences observed in ASD is the differential regulation of sex hormones and their receptor-mediated signaling in females and males, leading to differential gene transcription. In this review, we will discuss the possibility that regulation of inflammation by sex hormone nuclear receptors (NRs) contributes to the observed sex differences in ASD.

Though both sex differences and immune involvement are well established features of ASD, mechanisms linking sex and immune factors in neurodevelopmental disorders like ASD are not as well studied. However, the importance of sex in inflammation has been demonstrated in other biological contexts. Sex-dependent inflammatory phenotypes are observed in response to innate and adaptive immune reactions as well as in acute and chronic inflammatory diseases and their animal models (Klein and Flanagan, 2016; Chamekh and Casimir, 2019; Gal-Oz et al., 2019). Males are generally more susceptible to pathogen infections (Klein, 2012; Vazquez-Martinez et al., 2018), while females are more often diagnosed with autoimmune diseases (Quintero et al., 2012; Ngo et al., 2014; Billi et al., 2019; Lasrado et al., 2020). For example, in experimental autoimmune encephalomyelitis, a mouse model of multiple sclerosis, female and male mice have differing disease courses (Constantinescu et al., 2011). Phenotypes also differ by sex in animal models of high-fat diet, which induces low grade but chronic inflammation in macrophages and disrupts homeostasis in adipose tissues, resulting in induction of metabolic syndrome (Lumeng et al., 2007; Duan et al., 2018). Male mice gain weight and display insulin resistance, while female mice are more resistant to these effects (Pettersson et al., 2012; Ingvorsen et al., 2017; Casimiro et al., 2021). These observations suggest that sex-specific factors are important in regulating inflammation.

### MIA-Induced Inflammation as a Model of ASD

**FIGURE 1:**
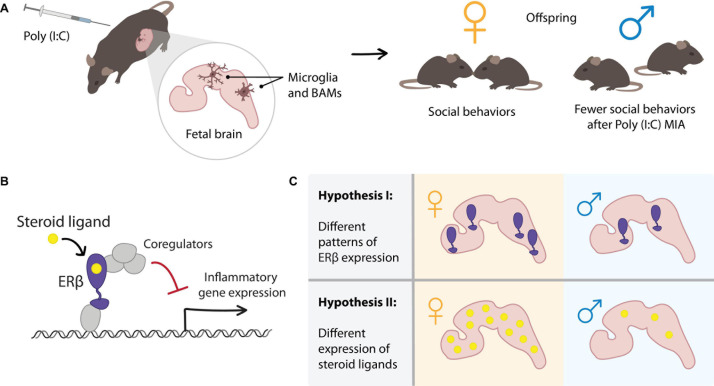
Hypothesized role of estrogen receptor (ER)β signaling in mediating sex differences in the maternal immune activation (MIA) mouse model of ASD. **(A)** In the MIA model, polyinosinic:polycytidylic acid [Poly(I:C)] is injected into wild-type pregnant female mice at E12.5. MIA results in inflammatory signaling, including responses in myeloid-lineage cells in the fetal brain (microglia and BAMs). Offspring of Poly(I:C) treated dams display sex-specific behavioral phenotypes such as decreased social interaction in male offspring but not in female offspring. To explain the sex difference in the MIA-induced ASD mouse model, we consider sex steroid hormone nuclear receptor signaling in the fetal brain. **(B)** Mechanism of ERβ-mediated repression of inflammatory gene expression, which we previously observed in microglia. **(C)** Two hypothesized mechanisms by which sex differences in ERβ signaling in the fetal brain could contribute to the sex differences observed in the MIA model. Differential expression of (I) ERβ or (II) steroid ligands in female and male fetal brains could result in differential transcriptional responses to the MIA inflammatory stimulus.

The maternal immune activation (MIA)-induced animal model of ASD has the potential to reveal insights about the impact of sex-specific and immune factors, and their interactions, during brain development. The MIA model was developed based on the observation that infection during pregnancy is linked to ASD (Atladottir et al., 2010; Zerbo et al., 2015; Al-Haddad et al., 2019). Outbreaks of several viruses, such rubella and influenza, have been documented to be associated with increased numbers of individuals with ASD (Zerbo et al., 2013; Shuid et al., 2021). Consistent with these findings, the MIA model uses the injection of a toll-like receptor (TLR) ligand into pregnant wild-type female mice on a specific day of gestation to induce an immune response. A commonly used ligand is polyinosinic:polycytidylic acid [Poly(I:C)], which mimics infection by double-stranded RNA viruses and triggers the TLR3-mediated innate immune response (Smith et al., 2007; Patterson, 2011). This MIA-induced ASD model displays behavioral phenotypes, including decreased sociability, increased repetitive restricted behavior, impaired learning and memory, altered levels of anxiety, and hyperactivity (Patterson, 2011; Estes and McAllister, 2016). Importantly, several groups have reported that the behavioral phenotypes in this model are only observed in male offspring (Xuan and Hampson, 2014; Coiro and Pollak, 2019; Haida et al., 2019; Keever et al., 2020; Nichols et al., 2020, preprint; Figure 1A). Based on these findings, MIA induction in mice is widely used to study the mechanism of ASD because it successfully recaptures behavioral phenotypes and sex-specific features observed in the disorder.

### Inflammation in Fetal Myeloid-Lineage Cells Upon MIA

It is currently hypothesized that maternal cytokines are the causative factor affecting fetal brain development in the MIA-induced model of ASD (Smith et al., 2007; Choi et al., 2016). Indeed, MIA induces an adaptive immune response in mothers, particularly the activation of a subset of T helper cells (Th17 T cells) and the release of maternal cytokines such as interleukin (IL)-17, that can affect fetal brain development in mice (Choi et al., 2016). However, a few groups, including ours, have reported that MIA may also directly induce an inflammatory innate immune response in fetal myeloid cells (Onore et al., 2014; Matcovitch-Natan et al., 2016; Carlezon et al., 2019; Ben-Yehuda et al., 2020; Cui et al., 2020; Nichols et al., 2020, preprint).

Brain myeloid-lineage cells derive from primitive macrophages in the yolk sac and migrate to the brain on embryonic day (E) 9.5 in mice, after which these cells expand, migrate, and develop into microglial cells and border-associated macrophages (BAMs) (Ginhoux et al., 2010; Goldmann et al., 2016; Utz et al., 2020). These two myeloid subsets have common as well as subset-specific gene expression profiles and localize to different areas of the brain: microglia in the brain parenchyma, and BAMs in the meninges and the choroid plexus (Ginhoux et al., 2010; Goldmann et al., 2016; Mrdjen et al., 2018; Jordao et al., 2019; Van Hove et al., 2019; Utz et al., 2020). A few studies point to BAMs as a key cell type in the response to MIA in the fetal brain. Although the precise mechanism is not clear, a recent publication indicates that MIA-activated BAMs in the choroid plexus secrete the chemokine CCL2 into the fetal ventricle, resulting in enhanced local inflammation (Cui et al., 2020). Moreover, our single-cell RNA-sequencing (scRNA-seq) analysis showed that the activation of fetal BAMs in response to MIA was dependent upon fetal *Trif*, an essential signaling molecule downstream of TLR3 (Nichols et al., 2020, preprint). These findings indicate that MIA leads to fetal innate immune signaling in BAMs. Furthermore, in validating our scRNA-seq data, we found that MIA causes BAMs in the choroid plexus, but not meningeal BAMs or microglia, to have increased expression of *S100a8 and 9*, key inflammatory genes that are known to induce chemotaxis and enhance inflammation (Ehrchen et al., 2009; Cesaro et al., 2012; Cury et al., 2013; Garcia-Arias et al., 2013; Walsham and Sherwood, 2016; Nishikawa et al., 2017; Aranda et al., 2018; Wang et al., 2019; Silvin et al., 2020). These data suggest that inflammation in fetal myeloid cells may be involved in the development of ASD-like changes in MIA-induced fetal brains. Furthermore, it is possible that differential regulation of this inflammation may be a mechanism to explain the sex-specific phenotypes observed in this mouse model.

### Expression of ERs and Sex Steroid Hormones in the Fetal Brain

Since MIA induces inflammation in brain myeloid-lineage cells, one hypothesis to explain the male bias in ASD is differing magnitude and duration of inflammation in males and females during fetal development. As we described above, in this review we will mainly discuss sex steroid NRs, especially ERβ, as potential regulators of fetal brain inflammation. We focus on ERβ because (1) ERβ is broadly expressed in mouse brain (Mitra et al., 2003; Fan et al., 2006) and (2) we previously showed that ERβ could regulate inflammation in microglial cells (Saijo et al., 2011).

So far, it is not clear whether ERα and ERβ expression in the myeloid cells of the fetal brain varies by sex. Studies have examined estrogen signaling primarily in whole brain or neuronal cells, and few have examined developmental time points prior to the neonatal period. Excellent reviews are available for overall brain expression analyses of ERα, ERβ, and enzymes required for the generation of androgens and estrogens (McCarthy, 2008; Bondesson et al., 2015). Several reports indicate that ERα, ERβ, and enzymes are present during mid-gestation. For example, ERβ expression was detected in the fetal midbrain, neuromere, hypothalamus, thalamus, and basal plate of pons at E12.5 (Fan et al., 2006), and ERα expression was observed at E16.5 in a gonadal sex dependent manner (Cisternas et al., 2015). In amygdala neuronal cultures obtained from E15 embryos, ERβ is sex-differentially regulated: lower levels of Esr2 mRNA expression were observed in females, but also sex differences in hormonal responsiveness were present, with increased Esr2 expression in response to 17β-estradiol or DHT hormonal stimulation only in females. These effects were dependent on sex chromosome complement (Cisternas et al., 2017). Activity of ERs, using an ERE-luciferase reporter, was observed in the fetal forebrain and hindbrain as early as E13.5, though no difference was detected between brains from females and males except in the P1 hindbrain (Della Torre et al., 2018). Several key enzymes involved in steroid hormone synthesis are expressed in female and male E16 fetal brain, including StAR, Cyp11a1, 5α-Reductase, and aromatase (Cisternas et al., 2015). Aromatase is an enzyme that converts testosterone to 17β-estradiol and androstenedione to estrone. Notably, sex-dependent expression of aromatase in the developing mouse brain has been reported, which may indicate the presence of differing concentrations of ER ligands in females and males that could impact downstream signaling (Harada and Yamada, 1992; Greco and Payne, 1994; Hutchison et al., 1997; Cisternas et al., 2015; Shay et al., 2018; Sellers et al., 2020).

Little is known about the expression of sex steroid hormones in the fetal mouse brain; however, a report showed that 17β-estradiol, testosterone, and DHT were detected in the brains of fetal mice, and that these hormones may exhibit sex dimorphic expression patterns in different brain regions (Konkle and McCarthy, 2011). However, to better understand how sex steroid hormones may regulate inflammation induced by MIA, precise analysis of sex steroid hormone expression in the fetal brain will be important.

Together, these expression studies suggest that the cellular machinery for ER signaling is present in the fetal brain from a relatively early age, and that sex differences in the expression of receptors, steroid metabolizing enzymes, and hormone ligands could contribute to differential regulation by ERs in females and males. Our favorite hypothesis is that concentrations of particular ER ligands differ between females and males in such a way that MIA-induced inflammatory responses differ in magnitude or duration. For example, ligands that induce transcriptional repression of inflammatory genes via ERβ may be highly expressed in female fetal brains, leading to efficient resolution of inflammation upon MIA. The hypothetically lower expression of such repressive ERβ ligands in fetal male brains could result in larger or prolonged inflammatory responses compared to females (Figure 1C, Hypothesis II). A comprehensive analysis of the expression of ERs and related ligands in developing fetal mouse brains, especially comparing sex, cell type, and specific brain region, will be important in understanding the contribution of ER-mediated transcription in sex-specific brain development.

### Nuclear Receptor Signaling in General

NRs are a family of transcription factors which both positively and negatively regulate transcription in response to ligand binding. Steroid hormone NRs are a class of NRs with activities that depend on endogenous small lipophilic ligands such as steroid hormones. For example, estrogen receptors (ERs) bind to estrogen response elements (essential ERE, 5’-GGTCAnnnTGACC-3’) (Driscoll et al., 1998; Klinge, 2001) in gene regulatory regions to control the expression of target genes. In addition to direct DNA binding, NRs can also regulate transcription by binding to other transcription factors *in trans*. NR function depends upon the ligands that are bound to the receptor. Indeed, NRs change their conformation in response to ligand binding in order to recruit either transcriptional activator or repressor complexes (Moras and Gronemeyer, 1998; Bourguet et al., 2000; Nagy and Schwabe, 2004), and it has been proposed that ligand binding may induce post-translational changes on NRs that stabilize co-factor binding (Hammer et al., 1999; Lannigan, 2003; Pascual et al., 2005; Lalevee et al., 2010; Anbalagan et al., 2012; Helzer et al., 2015; El Hokayem et al., 2017). To carry out their transcriptional activation and repression activities, NRs recruit a wide variety of co-factors and enzymes required for modifying histones and remodeling chromatin. These factors include histone acetyltransferases, deacetylases, methyltransferases, demethylases, and chromatin remolding factors, as well as kinases, phosphatases, and ubiquitin and SUMO E3 ligases (Olefsky, 2001; Perissi and Rosenfeld, 2005; Dasgupta et al., 2014).

### ERs and Their Impact on Inflammation

Various reports have suggested that sex steroid hormones and their steroid hormone nuclear receptors (NRs) may regulate inflammatory responses in innate immune cells. In particular, two estrogen receptor isoforms (ERα and ERβ) as well as the androgen receptor (AR) are well characterized sex steroid hormone NRs that are known to regulate innate immune responses (Vegeto et al., 2003; Baker et al., 2004; Suuronen et al., 2005; Harkonen and Vaananen, 2006; Sierra et al., 2008; Lai et al., 2009; Saijo et al., 2011; Kovats, 2015; Villa et al., 2015; Villa et al., 2016; Ardalan et al., 2019; Becerra-Diaz et al., 2020). We have previously reported that ERβ regulates the duration and magnitude of the inflammatory response in microglial cells (Saijo et al., 2011). ERβ binds a range of ligands, including estrogens and androgens, and specific ERβ ligands can facilitate repression of inflammation (Kuiper et al., 1997; Wu et al., 2013). See Figure 1B for a simplified schematic of ERβ-mediated transcriptional repression of inflammatory genes. Several reports have indicated that 17β-estradiol, a ligand for both ERα and ERβ, can regulate inflammation in myeloid-lineage cells. However, this regulation is not always clear in that some reports have suggested that ER-mediated transcription represses inflammation (Vegeto et al., 2003; Ribas et al., 2011), while others have suggested that it does not (Calippe et al., 2010; Shindo et al., 2020). While the amino acid sequences of the DNA-binding domains of these two ER isoforms are highly conserved, their ligand-binding domains (LBDs) are much less so (47% in human). Since the functions of NRs are dependent upon ligands, this lack of conservation in ER LBDs may suggest that ERα and ERβ may differ in their preferential ligands, and that binding of the same ligand to either ERα or ERβ could result in different transcriptional outputs.

Previously, we reported that ERβ represses inflammation in microglia in a ligand-dependent manner (Saijo et al., 2011). In mouse microglial cells, a subset of ligands, including the endogenous ligand 5-androsten-3β, 17β-diol (Δ5-Adiol) and the synthetic ligands Indazole-estrogen-Cl and -Br, have been shown to induce transcriptional repression of inflammation in an ERβ-dependent manner. Treatment with these repressive ligands, but not the classic ER ligand 17β-estradiol, results in the recruitment of the transcriptional corepressor CtBP (Saijo et al., 2011; Figure 1B). CtBP is a co-repressor platform that is known to assemble enzymes required for transcriptional repression, such as euchromatic histone-lysine N-methyltransferase 2 (EHMT2, also known as G9a), euchromatic histone-lysine N-methyltransferase 1 (EHMT1, also known as GLP), the histone deacetylases HDAC1 and 2, and lysine demethylase 1A (KDM1a, also known as LSD1) (Chinnadurai, 2002; Dcona et al., 2017). When microglial cells are stimulated with the TLR4 ligand lipopolysaccharide (LPS), ERβ binds to cFos and repressive ligands, which results in the recruitment of the CtBP complex to target genes, thus regulating inflammation through a transrepression mechanism. Interestingly, mutations in ERβ, CtBP1/2, and HDACs have been observed in human ASD patients (Chakrabarti et al., 2009; Zettergren et al., 2013; De Rubeis et al., 2014). Although these NRs and their co-factors/binding partners are proposed to be genetic factors for ASD, we consider the possibility that these steroid hormone NRs and their ligands may exert their effects on brain development by modulating the inflammatory response to environmental immune stimuli.

## Conclusion and Future Directions

Endocrine disruption, such as sex hormone dyshomeostasis, during fetal brain development increases the risk of NDDs (Colborn, 2004; Schug et al., 2015; Moosa et al., 2018). Further supporting the role of sex hormone signaling in brain development, ERβ conventional knockout mice show fewer proliferating cells and more apoptotic cells in the E18.5 fetal brain (Wang et al., 2003). These observations underscore the importance of sex hormone nuclear receptor-mediated signaling during brain development in addition to the well-known role of hormone signaling in sex differentiation of the brain. Investigating the role of ER signaling in different cell types and across developmental time periods will clarify the mechanisms underlying the observed brain phenotypes after disruption of hormone signaling pathways.

Here, we have discussed the hypothesis that ERβ-mediated repression of inflammation in brain myeloid-lineage cells may contribute to the male bias observed in an MIA-induced ASD mouse model. We consider two hypotheses of how ERβ-mediated transcription may contribute to the sex-specific phenotypes in the MIA model. One is that the expression of ERβ may be different between fetal female and male brains. The other is that ERβ ligands that induce transcriptional repression may differ in fetal female and male brains (Figure 1C). Therefore, a precise mechanistic understanding of ERβ-mediated transcription and a thorough analysis of the expression of sex steroid hormones and their receptors in the brain may provide new insights into the sex-dependent phenotypes in ASD and other neurodevelopmental disorders.

## Author Contributions

MA and KS wrote the manuscript. MA made the figure. Both authors contributed to the article and approved the submitted version.

## Author Disclaimer

Any opinions, findings, and conclusions or recommendations expressed in this material are those of the author(s) and do not necessarily reflect the views of the National Science Foundation.

## Conflict of Interest

The authors declare that the research was conducted in the absence of any commercial or financial relationships that could be construed as a potential conflict of interest.

## Publisher’s Note

All claims expressed in this article are solely those of the authors and do not necessarily represent those of their affiliated organizations, or those of the publisher, the editors and the reviewers. Any product that may be evaluated in this article, or claim that may be made by its manufacturer, is not guaranteed or endorsed by the publisher.
